# Supplementary material for: The Chromatin-Oxygen Sensor Gene KDM5C Associates with Novel Hypoxia-Related Signatures in Glioblastoma Multiforme
Source: Int J Mol Sci. 2022 Sep 6;23(18):10250. doi: 10.3390/ijms231810250 (PMC9498997; doi:10.3390/ijms231810250)
Supplement: Supplementary file 1 [file ijms-23-10250-s001.zip › Supplementary Data.pdf]

SUPPLEMENTARY DATA

Supplementary Materials and Methods

RNA isolation and quantitative real-time PCR. Total RNA was isolated from GBM biopsies using TRIzol Reagent (Invitrogen, Milan, Italy). Briefly, samples were homogenized in TRIzol reagent using Tissue Lyser LT (Qiagen, Hilden, Germany). Total RNA extraction and DNAse treatment were performed according to the manufacturer's protocols (Life Technologies, Carlsbad, CA, USA). Reverse transcription was performed with Superscript III Reverse Transcription kit (Life Technologies, Carlsbad, CA, USA). Quantitative real-time PCR were performed by using iTaq Universal Master Mix BioRad Laboratories Inc., Hercules, USA) in the CFX96 touch real time detection system (BioRad Laboratories Inc., Hercules, USA). The primers used are listed in Supplementary data (Table S4). *18S* and Tata-Binding Protein (*TBP*) were used as reference genes. Each experiment assay was performed in triplicate in three independent experiments.

DNA preparation and high-resolution melting analysis. Tumor DNA was isolated from frozen tissues using a QIAamp DNA Mini Kit (Qiagen, Tokyo, Japan). For the *IDH1/2* mutational analysis, high-resolution melting (HRM) reactions were performed by using Precision Melt Supermix (BioRad Laboratories Inc., Hercules, USA) and specific oligonucleotides elsewhere reported [80]. A CFX 96 Opus (BioRad Laboratories Inc., Hercules, USA) was used for amplification by applying the cycling conditions elsewhere described [80].

Bisulfite conversion and MGMT promoter methylation. Bisulfite conversion was done by using Ampli-MGMT Kit (Dia-chem S.r.l.) according to the manufacturers' protocol. Promoter methylation analysis of O6-methylguanine-DNA methyltransferase (*MGMT)* gene was carried out with Ampli-MGMT Kit (Dia-chem S.r.l.) according to the manufacturers' protocol.

Data collection and bioinformatics analysis. Expression and methylation data of the GBM cohort (Firehose Legacy) of The Cancer Genome Atlas (TCGA) Consortium were retrieved from the public repository cBioportal (https://www.cbioportal.org/). Only for 528 samples (out of 619) the expression data (U133 microarray) were available. Due to the lack of an appropriate number of normal control samples in the GBM-TCGA cohort (N=5), to verify if KDM5C gene is differentially expressed in the comparison tumor vs healthy we used expression data of post-mortem brains from The Genotype-Tissue Expression (GTEx; https://gtexportal.org/home/). We used a comparison tool available on GEPIA database (http://gepia.cancer-pku.cn/index.html) to generate the plot of Figure 4A. Afterwards, GBM samples were stratified according to the normalized expression values of *KDM5C* gene. Those displaying KDM5C levels ranking in the fourth quartile (i.e. the highest 25% of *KDM5C* gene intensity) were marked as KDM5C^{High} (N=132); conversely, those ranking in the first quartile (i.e. the lowest 25% of *KDM5C* gene intensity) were labelled as KDM5C^{Low} (N=132). Differentially expressed genes (DEGs) were then identified by comparing microarray data of these two tumor subgroups (generate on cBioportal). Only genes having qvalues (multiple correction derived from Benjamini-Hochberg procedure) <0.05 were marked as DEGs and selected for further analyses. Likewise, DNA Methylation (HM-27) data from the same GBM cohort (TCGA) - probe intensities values on the whole gene body - were downloaded from cBioportal and were used to identify differentially methylated genes between the two subgroups. Notably, HM27 beta-values were available only for 285 cases. As indicated in cBioportal database, for genes having multiple methylation probes, the probe most anti-correlated with expression was chosen for the analysis. Only genes having qvalues (Benjamini-Hochberg correction) <0.05 were marked as differentially methylated and used for further analyses. Afterwards, considering the mean methylation values and expression levels of each independent gene, using Euler-Venn diagrams we classified the genes into clusters, focusing on those characterized either by hypomethylation and over-expression or hypermethylation and down-regulation in KDM5C^{High} (*vs* KDM5C^{Low}) subgroup. Hence, the identified DEGs and differentially methylated genes (DMGs) were further subjected to Gene Ontology (GO) analysis by using DAVID gene ontology analysis tool

(http://david.abcc. ncifcrf.gov/) and Panther classification system (www.pantherdb.org). Protein-protein interaction (PPI) network analysis was obtained using STRING (Search Tool for the Retrieval of Interacting Genes/Proteins; https://string-db.org) online database. The medium confidence (0.4) of the argument of interactions was set. The correlation analysis on gene expression data (RT-PCR) was performed using linear models implemented in R language (lmp function). Scatterplots showing the correlation of gene expression values were generated in R using ggplot2 library and custom scripts. Correlations between the selected genes were reported in the graphs as Pearson's coefficient (r) and p values were computed by one-way ANOVA in R. Cancer expression levels and matched clinical information were acquired from The Cancer Genome Atlas database ((https://genome-cancer.ucsc.edu/). The datasets were classified into two groups: expression datasets in tumor tissues and normal matched adjacent tissues. Using receiver operating characteristic (ROC) curve analysis, combined with sensitivity and specificity chosen as a cutoff point, the expression of *KDM5C* in GBM tumor patients was used to create two groups: the lower expression group and the higher expression group. Survival time of patients derived from the clinical dataset have been analyzed the difference between the lower and higher *KDM5C* expression groups and compared the 10-year overall survival rate by Kaplan-Meier analysis.

Protein extraction and Western Blotting (WB) analysis. Protein extraction was done in RIPA buffer by using Tissue Lyser LT (Qiagen, Hilden, Germany), as elsewhere described [81]. Membranes were blocked with 5% non-fat dry milk and the following antibodies were used: KDM5C (dilution 1:1000, ab8588, Abcam, Cambridge, UK). As loading controls, anti-β-ACTIN (dilution 1:5000, sc8432; Santa Cruz Biotechnology, Dallas, USA) was used. As secondary antibodies, anti-mouse (dilution 1:5000, sc-2005 Santa Cruz Biotechnology, Dallas, TX) and anti-rabbit (1:5000, sc-2004 Santa Cruz Biotechnology, Dallas, TX) were used. WB detection was done by using Enhanced Chemiluminescence Reagent Kit (ECL, Advansta, San Jose, USA).

Immunohistochemistry (IHC). Formalin-fixed paraffin-embedded tumor blocks were prepared as standard procedure. 4-µm-thick tissue sections were de-paraffinized in xylene and hydrated by immersing in a series of graded ethanol. Quantitative analysis of p53 and Ki67 proteins were done by IHC with the relative antibodies against p53 (anti-p53; Bp53-11, Ventana Medical Systems, Roche Diagnostics) and Ki67 (anti-Ki67; 30-9, Ventana Medical Systems, Roche Diagnostics), following the standard procedures. The IHC staining was developed with UltraView Universal DAB-Detection Kit on the BenchMark Ultra (Ventana Medical Systems, Roche Diagnostics). For the immunohistochemical expression analysis, the nuclear staining of p53 and Ki67 were scored semi-quantitatively in the most prominently stained area of the tissue slides. p53 expression was reported as negative, weak, moderate or strong when <1%, 1-10%, 10-50% or ≥ of GBM tumor cells displayed p53 immunoreactivity, respectively.

Statistical analysis. One-way ANOVA with Dunn multiple comparisons test, One-way ANOVA with Friedmans multiple comparisons and Student's t-test were applied using GraphPad Prism 7 software (GraphPad Software, La Jolla, CA, USA). Data are expressed as mean ± SEM. For Survival data, the prognostic significance analysis was performed using Kaplan-Meier method and log-rank tests. The clinical variables between the two KDM5C subgroups were examined by chi-square test. Experiments were performed a minimum of three independent times and always performed in independent triplicate samples. Results were statistically significant in case of $p<0.05$.

Supplementary Reference List

80. Hatae, R.; Hata, N.; Yoshimoto, K.; Kuga, D.; Akagi, Y.; Murata, H.; Suzuki, S. O.; Mizoguchi, M.; Iihara, K. Precise Detection of IDH1/2 and BRAF Hotspot Mutations in Clinical Glioma Tissues by a Differential Calculus Analysis of High-Resolution Melting Data. *PloS one.* **2016**, *11*, e0160489. doi.org/10.1371/journal.pone.0160489.
81. Bien-Möller, S.; Balz, E.; Herzog, S.; Plantera, L.; Vogelgesang, S.; Weitmann, K.; Seifert, C.; Fink, M.A.; Marx, S.; Bialke, A. et al. Association of Glioblastoma Multiforme Stem Cell

Characteristics, Differentiation, and Microglia Marker Genes with Patient Survival. *Stem cells international.* **2018**, 9628289. doi.org/10.1155/2018/9628289.

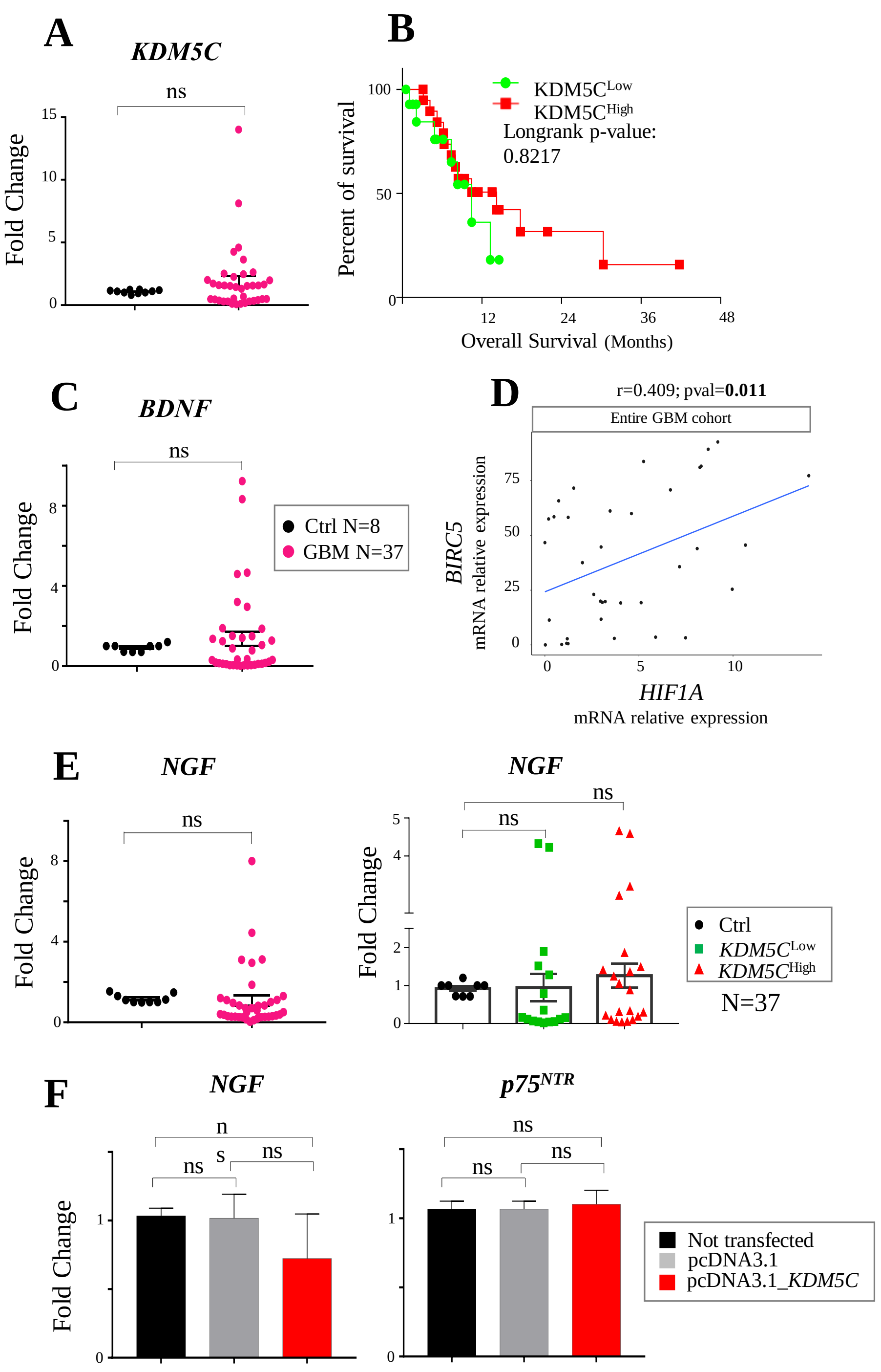
A
KDM5C
ns
Fold Change
B
KDM5C^Low
KDM5C^High
Longrank p-value:
0.8217
Percent of survival
Overall Survival (Months)
C
BDNF
ns
Fold Change
Ctrl N=8
GBM N=37
D
r=0.409; pval=0.011
Entire GBM cohort
BIRC5
mRNA relative expression
HIF1A
mRNA relative expression
E
NGF
ns
Fold Change
NGF
ns
ns
Fold Change
Ctrl
KDM5C^Low
KDM5C^High
N=37
F
NGF
ns
ns
ns
Fold Change
p75^NTR
ns
ns
ns
Not transfected
pcDNA3.1
pcDNA3.1_KDM5C

Fig S1

A

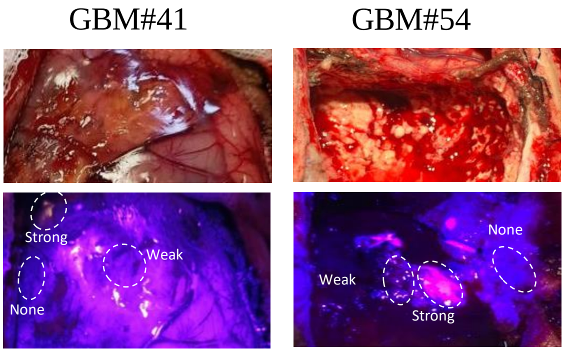
GBM#41
GBM#54
Strong
Weak
None
None
Weak
Strong

B

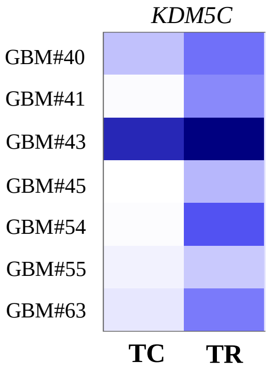
KDM5C
GBM#40
GBM#41
GBM#43
GBM#45
GBM#54
GBM#55
GBM#63
TC
TR

Fig S2

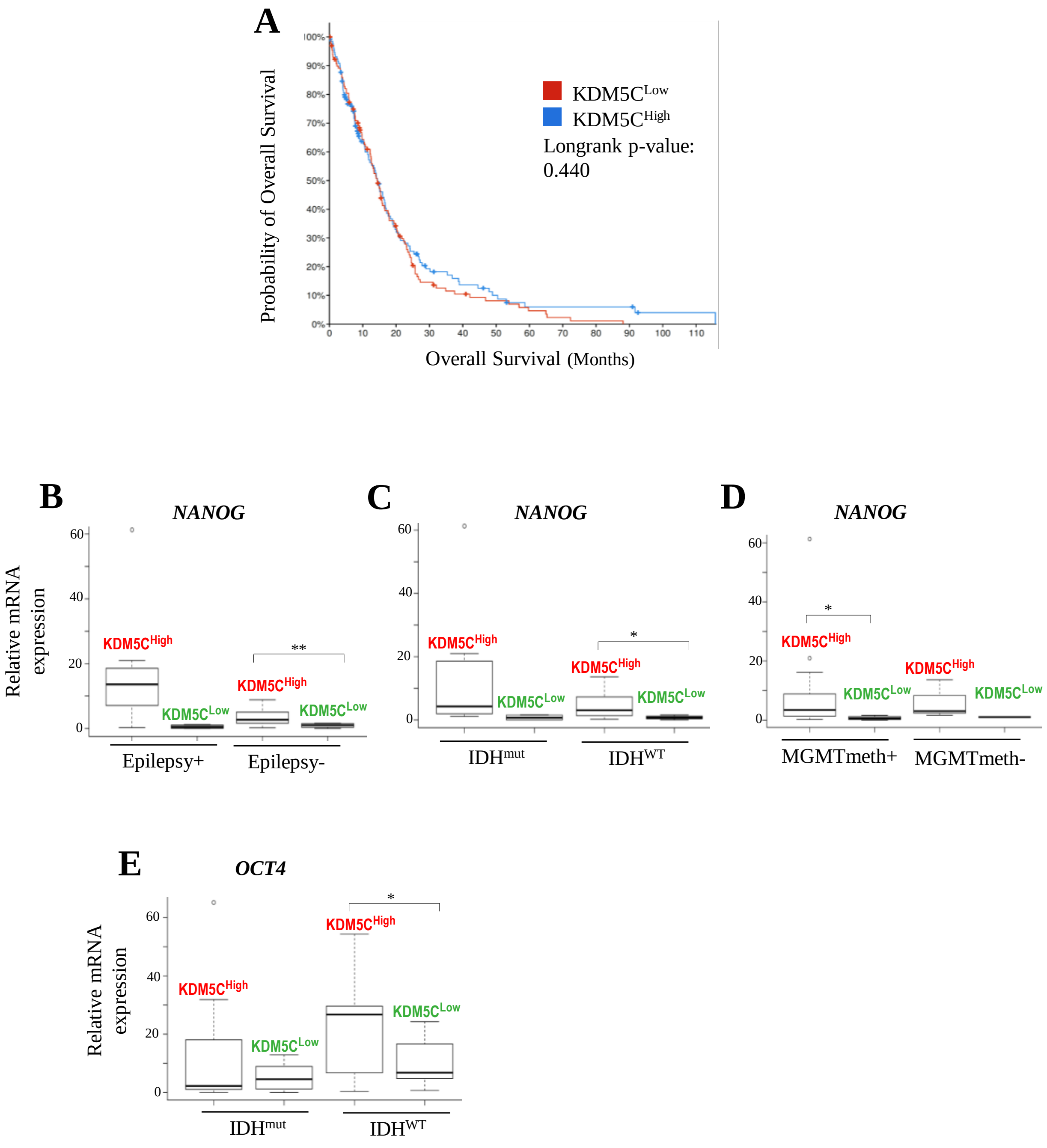
A
Probability of Overall Survival
100%
90%
80%
70%
60%
50%
40%
30%
20%
10%
0%
0
10
20
30
40
50
60
70
80
90
100
110
KDM5C^Low
KDM5C^High
Longrank p-value:
0.440
Overall Survival (Months)
B
NANOG
Relative mRNA expression
60
40
20
0
KDM5C^High
KDM5C^Low
KDM5C^High
KDM5C^Low
**
Epilepsy+
Epilepsy-
C
NANOG
60
40
20
0
KDM5C^High
KDM5C^Low
KDM5C^High
KDM5C^Low
*
IDH^mut
IDH^WT
D
NANOG
60
40
20
0
*
KDM5C^High
KDM5C^Low
KDM5C^High
KDM5C^Low
MGMTmeth+
MGMTmeth-
E
OCT4
Relative mRNA expression
60
40
20
0
KDM5C^High
KDM5C^Low
*
KDM5C^High
KDM5C^Low
IDH^mut
IDH^WT

Fig S3

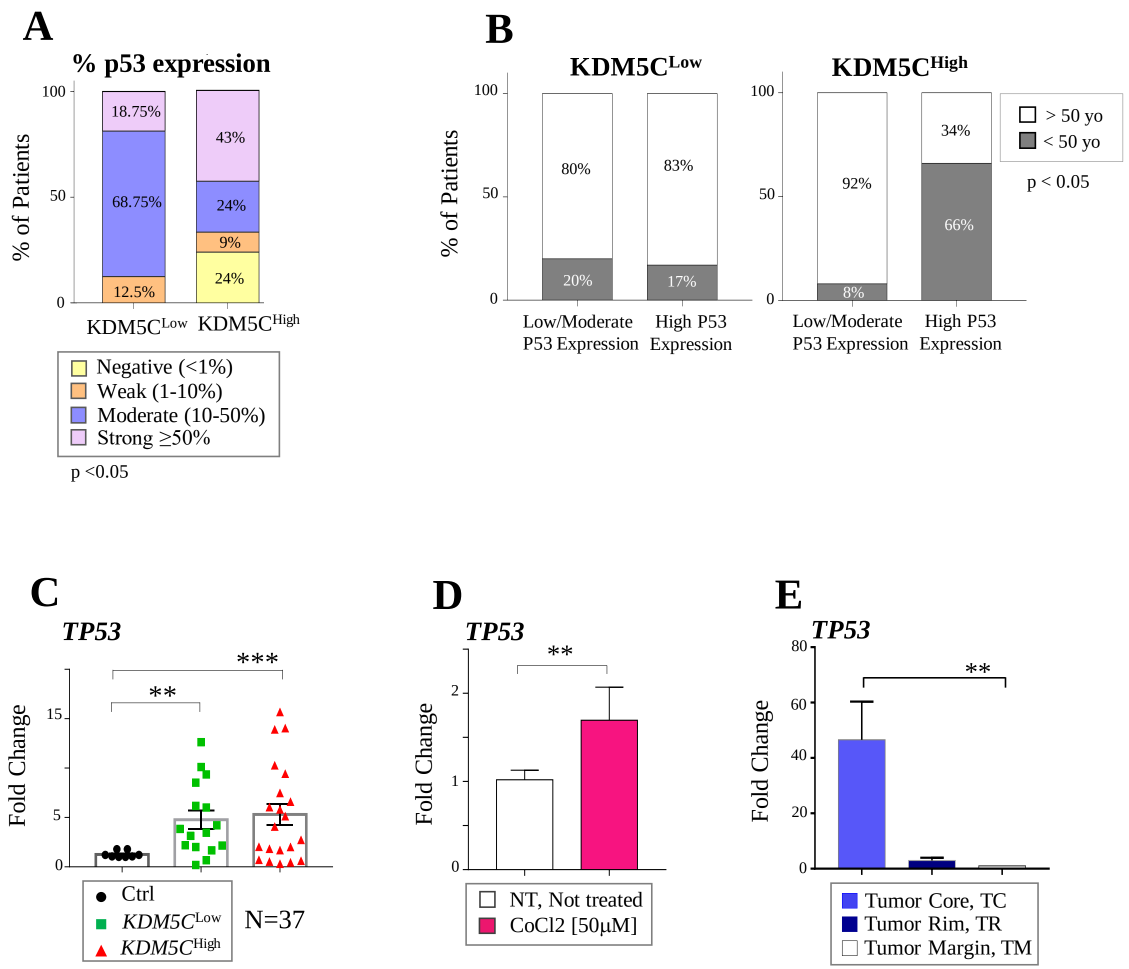
A
% p53 expression
% of Patients
100
50
0
18.75%
68.75%
12.5%
43%
24%
9%
24%
KDM5CLow
KDM5CHigh
Negative (<1%)
Weak (1-10%)
Moderate (10-50%)
Strong ≥50%
p <0.05
B
KDM5CLow
KDM5CHigh
% of Patients
100
50
0
80%
20%
83%
17%
92%
8%
34%
66%
Low/Moderate P53 Expression
High P53 Expression
> 50 yo
< 50 yo
p < 0.05
C
TP53
Fold Change
15
5
0
**
***
Ctrl
KDM5CLow
KDM5CHigh
N=37
D
TP53
Fold Change
2
1
0
**
NT, Not treated
CoCl2 [50µM]
E
TP53
Fold Change
80
60
40
20
0
**
Tumor Core, TC
Tumor Rim, TR
Tumor Margin, TM

Fig S4

Legend to Supplementary Figures

Figure S1. (**A**) Real time PCR (RT-PCR) assay of *KDM5C* levels in the entire cohort of GBM patients ($KDM5C^{Tot}$). (**B**) Kaplan–Meier curves of the survival of patients with $KDM5C^{Low}$ and $KDM5C^{High}$. (**C**) RT-PCR assay of *BDNF* levels in $KDM5C^{Tot}$ patients. (**D**) Scatter plot reporting the correlation - by linear regression analysis - of *HIF1A* and *BIRC5* expression in the entire GBM cohort. (**E**) RT-PCR assay of NGF in the in the entire GBM cohort (*left panel*) and in the $KDM5C^{Low}$ and $KDM5C^{High}$ subgroups (*right panel*). (**F**) RT-PCR analysis of NGF and $p75^{NTR}$ expression in T98G cell lines transiently transfected with a plasmid expressing *KDM5C* or a plasmid containing the empty vector. Each transcript analysis was performed in triplicate and the samples were normalized with TATA-Box Binding Protein (*TBP*) transcript. The bars indicate the mean ± sem of repeated experiments. ns, not significant.

Figure S2. (**A**) Representative intraoperative photographs showing grading of 5-ALA fluorescence intensities. Photograph of tumor brain parenchyma under normal light conditions (*upper panels*) and illuminated with blue light (*bottom panels*). (**B**) Heat map illustrating differences of *KDM5C* expression in TC and TR niches.

Figure S3. (A) Kaplan–Meier curves of the survival of TCGA patients with $KDM5C^{Low}$ and $KDM5C^{High}$; (**B**) *NANOG* expression analysis in GBM patients with or without epilepsy; (**C**) in GBM patients with wild-type or mutated *IDH1/2* alleles, and (**D**) in GBM patients with or without *MGMT* methylation; (**E**) *OCT4* expression analysis in GBM patients with wild-type or mutated *IDH1/2* alleles. In the box plot, the black thick line inside the box represents the median of the values and 25th and 75th percentile are shown as boundaries of the box. Student t-test was applied with **$p<0.005$, *$p<0.05$.

Figure S4. (**A**) % of p53 protein expression in the four p53 expression subgroups in KDM5C^{Low} and KDM5C^{High} patients; (**B**) % of early and late GBM onset in KDM5C^{Low} and KDM5C^{High} patients with low/moderate or high p53 protein expression; (**C-E**) RT-PCR assay of *TP53* levels in KDM5C^{Low} and KDM5C^{High} subgroups, in T98G cell lines exposed to cobalt chloride ($CoCl_2$), and in 5-ALA FGS tissues. The bars indicate the mean ± SEM or Dev.St. of repeated experiments. Asterisks indicate statistical significance compared with control sample, ***$p<0.0005$, **$p< 0.005$.
