# Supplementary material for: The Chromatin-Oxygen Sensor Gene KDM5C Associates with Novel Hypoxia-Related Signatures in Glioblastoma Multiforme
Source: Int J Mol Sci. 2022 Sep 6;23(18):10250. doi: 10.3390/ijms231810250 (PMC9498997; doi:10.3390/ijms231810250)
Supplement: Supplementary file 1 [file ijms-23-10250-s001.zip › Table S1.pdf]

Table S1. Clinico-pathological characteristics of *KDM5C*^{Low} and *KDM5C*^{High} cohorts.

GBM cohort	Age (Years) No; %		Gender		Tumor location (Hemisphere)			Lesion number		Epilepsy		MGMT methylation		IDH1/2 mutation		Chemotherapy		Radiotherapy		Survival rate
	<50	>50	M	F	Right	Left	Others	S	M	Yes	No	Yes	No	WT	Mut	Yes	No	Yes	No	
***KDM5C*^{Low}**	5<br>13.5%	11<br>29.7%	9<br>24.3%	7<br>19%	7<br>19%	6<br>16.4%	3<br>8%	13<br>35%	3<br>8%	6<br>16.2%	10<br>27%	15<br>40.5%	1<br>2.7%	6<br>16.2%	10<br>27.1%	14<br>38%	2<br>5.3%	11<br>30%	5<br>13.5%	53%
***KDM5C*^{High}**	5<br>13.5%	16<br>43.3%	11<br>29.7%	10<br>27%	10<br>27%	11<br>29,7%	0	20<br>54%	1<br>3%	7<br>18.9%	14<br>37.9%	18<br>48.8%	3<br>8%	8<br>21.6%	13<br>35.1%	20<br>54%	1<br>2.7%	18<br>48.5%	3<br>8%	40%
Total No	10<br>27%	27<br>73%	20<br>54%	17<br>46%	17<br>45.9%	17<br>45.9%	3<br>8%	33<br>89%	4<br>11%	13<br>35%	24<br>65%	33<br>89.1%	4<br>10.9%	14<br>38%	23<br>62%	34<br>92%	3<br>8%	29<br>78.3%	8<br>21.7%	
p-value (Chi Square test)	0.65		0.81		0.72			0.29		0.99		0.43		0.39		0.56		0.76		0.82

M, male; F, female; WT, wild-type; Mut, Mutation; +, positive; -, negative; S, single; M, multifocal; p-value <0.05
