# Supplementary material for: The Chromatin-Oxygen Sensor Gene KDM5C Associates with Novel Hypoxia-Related Signatures in Glioblastoma Multiforme
Source: Int J Mol Sci. 2022 Sep 6;23(18):10250. doi: 10.3390/ijms231810250 (PMC9498997; doi:10.3390/ijms231810250)
Supplement: Supplementary file 1 [file ijms-23-10250-s001.zip › Table S4.pdf]

Table S4. Oligonucleotides used in this study.

Gene	Forward (F) and Reverse (R) Sequence 5’ to 3’
KDM5C	F_CTCCTTGCTACGCTCCCACTACGA
	R_TCAAATGGACGTGTGTTACAC
BDNF	F_ACACAAAAGAAGGCTGCAGG
	R_TGCTATCCATGGTAAGGGCC
NGF	F_GAGCAGCTTTCTATCCTGGC
	R_TTCCGCCTGTATGCCGATC
P75	F_CTGTGGTTGTGGGCCTTGT
	R_GCTGTCCACGGAGATGCC
HIF1A	F_TCCATGTGACCATGAGGAAA
	R_AAGCTTCGCTGTGTGTTTTG
NESTIN	F_GCGGGCTACTGAAAAGTTCC
	R_GCTGAGGGACATCTTGAGGT
OCT4	F_TTCGCAAGCCCTCATTTCAC
	R_CCATCACCTCCACCACCTG
SOX2	F_GCCGAGTGGAAACTTTTGTCG
	R_GGCAGCGTGTACTTATCCTTCT
NANOG	F_AGAAGGCCTCAGCACCTAC
	R_GGCCTGATTGTTCCAGGA
SURVIVIN	F_GGACCACCGCATCTCTACAT
	R_GAAACACTGGGCCAAGTCTG
TREM2	F_ CTCTCCGGCTGCTCATCTTA
	R_TGGAGTCATAGGGGCAAGAC
IL-33	F_TGGCCTACCATCCCTTCTGACCC
	R_CGTTTTGGCATCCAGAGGTCAGGT
IL-10	F_CCCAGACATCAAGGCGCATGTG
	R_GTAGATGCCTTTCTCTTGGAGC
IL-6	F_ CCCCTGACCCAACCACAAAT
	R_ ATTTGCCGAAGAGCCCTCAG
P53	F_CCTCAGCATCTTATCCGAGTGG
	R_TGGATGGTGGTACAGTCAGAGC
TBP	F_GAGCTGTGATGTGAAGTTTCC
	R_TCTGGGTTTGATCATTCTGTAG
18S	F_TCCCCATGAACGAGGAATTC
	R_GGCCTCACTAAACCATCCAA
IDH-1	F_GGCTTGTGAGTGGATGGGTA
	R_GCAAAATCACATTATTGCCAAC
IDH-2	F_AGCCCATCATCTGCAAAAAC
	R_CTCCACCCTGGCCTACCT
